# Efficacy Projection of Obiltoxaximab for Treatment of Inhalational Anthrax across a Range of Disease Severity

**DOI:** 10.1128/AAC.00972-16

**Published:** 2016-09-23

**Authors:** Brent J. Yamamoto, Annette M. Shadiack, Sarah Carpenter, Daniel Sanford, Lisa N. Henning, Edward O'Connor, Nestor Gonzales, John Mondick, Jonathan French, Gregory V. Stark, Alan C. Fisher, Leslie S. Casey, Natalya V. Serbina

**Affiliations:** a Elusys Therapeutics, Inc., Pine Brook, New Jersey, USA; b Battelle, West Jefferson, Ohio, USA; c Metrum Research Group LLC, Tarrifville, Connecticut, USA; d BDM Consulting, Inc., Somerset, New Jersey, USA

## Abstract

Inhalational anthrax has high mortality even with antibiotic treatment, and antitoxins are now recommended as an adjunct to standard antimicrobial regimens. The efficacy of obiltoxaximab, a monoclonal antibody against anthrax protective antigen (PA), was examined in multiple studies conducted in two animal models of inhalational anthrax. A single intravenous bolus of 1 to 32 mg/kg of body weight obiltoxaximab or placebo was administered to New Zealand White rabbits (two studies) and cynomolgus macaques (4 studies) at disease onset (significant body temperature increase or detection of serum PA) following lethal challenge with aerosolized Bacillus anthracis spores. The primary endpoint was survival. The relationship between efficacy and disease severity, defined by pretreatment bacteremia and toxemia levels, was explored. In rabbits, single doses of 1 to 16 mg/kg obiltoxaximab led to 17 to 93% survival. In two studies, survival following 16 mg/kg obiltoxaximab was 93% and 62% compared to 0% and 0% for placebo (*P* = 0.0010 and *P* = 0.0013, respectively). Across four macaque studies, survival was 6.3% to 78.6% following 4 to 32 mg/kg obiltoxaximab. In two macaque studies, 16 mg/kg obiltoxaximab reduced toxemia and led to survival rates of 31%, 35%, and 47% versus 0%, 0%, and 6.3% with placebo (*P* = 0.0085, *P* = 0.0053, *P* = 0.0068). Pretreatment bacteremia and toxemia levels inversely correlated with survival. Overall, obiltoxaximab monotherapy neutralized PA and increased survival across the range of disease severity, indicating clinical benefit of toxin neutralization with obiltoxaximab in both early and late stages of inhalational anthrax.

## INTRODUCTION

Clinical management of inhalational anthrax remains a topic of interest and concern in the United States (1, 2). In addition to anthrax outbreaks resulting from intentional release of spores as a bioterrorist weapon, accidental exposures may lead to inhalational anthrax cases (3–7). Inhalational anthrax is often fatal, despite treatment with antibiotics, because of rapid progression to bacteremia and toxemia (8, 9). In the 2001 U.S. anthrax attacks, inhalational anthrax had a fatality rate of 45% despite aggressive treatment with antibiotics and supportive therapy (10).

The pathogenesis of inhalational anthrax is driven by a tripartite toxin complex composed of the enzymatic moieties lethal factor (LF) and edema factor (EF) and a common cell-binding component, protective antigen (PA) (11–13). Neutralization of PA is an effective treatment and prevention strategy (14), and antitoxins are recommended by the U.S. Centers for Disease Control and Prevention for use in patients with a high level of suspicion for systemic anthrax in conjunction with appropriate antimicrobial therapy (1, 15).

Obiltoxaximab (ETI-204), a chimeric IgG1(κ) monoclonal antibody, prevents binding of PA to the receptors (16–18) and was recently licensed under the U.S. Food and Drug Administration's (FDA's) Animal Rule (Code of Federal Regulations 21 CFR 601.90) for treatment of inhalational anthrax at a therapeutic dose of 16 mg/kg of body weight administered intravenously in combination with appropriate antibacterial drugs. Obiltoxaximab is the second monoclonal antibody approved for treatment of inhalational anthrax. Other FDA-approved antitoxin therapies indicated for treatment of inhalational anthrax include a monoclonal antibody, raxibacumab (19, 20), and a polyclonal anthrax immune globulin, anthrasil (21).

Obiltoxaximab efficacy was evaluated in two well-characterized animal models for inhalational anthrax, New Zealand White (NZW) rabbit (22) and cynomolgus macaque (23), in studies designed to mimic human clinical trials. Results of two rabbit and four cynomolgus macaque studies are presented here. Survival data were integrated with a modeling approach to understand the impact of disease progression on obiltoxaximab-mediated survival and to predict the monotherapy window of effectiveness.

## MATERIALS AND METHODS

### Test system.

NZW rabbits (Oryctolagus cuniculus, specific pathogen free), weighing 2.9 to 4.0 kg and aged 0.6 to 1.6 years with implanted vascular access ports (VAPs), were procured from Covance Research Products, Inc. (Denver, PA).

Cynomolgus macaques (Macaca fascicularis) weighing 2.2 to 5.3 kg and aged 2.6 to 5.1 years were procured from Covance, Inc. (Alice, TX). Macaques were verified negative for intestinal parasites and tuberculosis, were seronegative for simian immunodeficiency virus, simian T-lymphotrophic virus-1, and Cercopithecine herpesvirus 1, and were negative for simian retrovirus (SRV-1 and SRV-2). Only healthy macaques free of malformations and clinical signs of disease were placed on study. Anti-PA IgG was conducted as a prescreen (inclusion criteria) for study M4 only. The testing was conducted at the supplier (Covance) with a total of 74 primates prescreened for anti-PA IgG prior to being placed in the study. One animal tested positive and was not entered in the study.

### Design of studies.

The studies were randomized, placebo-controlled, and parallel group (50% male, 50% female) with a range of obiltoxaximab doses. Two studies were conducted in rabbits in 2008 and 2011 (Table 1, R1 and R2) and four in cynomolgus macaques between 2009 and 2014 (Table 1, M1 to M4). Animals in the treatment studies, except for study M4, were randomized to treatment before challenge with B. anthracis spores. Animals were grouped into three strata based on weight, with equal numbers of male and female animals in each group. Separate randomizations to treatment were conducted within each of the strata. In study M4, treatment vials were randomized and animals were assigned to vials as they triggered for treatment. Rabbits or macaques were exposed (nose only or head only, respectively) to an aerosolized dose of B. anthracis spores (Ames strain) targeting 200 times the median lethal dose (24, 25) by real-time plethysmography.

**TABLE 1:** Overview of studies conducted with obiltoxaximab

Animal and study	Yr conducted	Study arm		Total no. of animals^*a*^	No. (%) of animals with trigger by^*c*^:		
		Obiltoxaximab (mg/kg)	Levofloxacin		PA-ECL	SIBT	Time
NZW rabbits							
R1	2008	0		9	5 (55.6)	4 (44.4)	0
		1		9	6 (66.7)	3 (33.3)	0
		4		17	6 (35.3)	11 (64.7)	0
		16		17	8 (47.1)	9 (52.9)	0
			50 mg/kg/day per os for 5 days	10	6 (60)	4 (40)	0
R2	2011	0		14	2 (14.3)	12 (85.7)	0
		1		14	3 (21.4)	11 (78.6)	0
		4		14	5 (35.7)	9 (64.3)	0
		8		14	5 (35.7)	9 (64.3)	0
		16		14	2 (14.3)	12 (85.7)	0
Cynomolgus macaques							
M1	2009	0		14	13 (92.9)	NA	1 (7.1)
		4		14	12 (85.7)	NA	2 (14.3)
		8		15	15 (100)	NA	0
M2	2010	0		16	16 (100)	NA	0
		4		16	16 (100)	NA	0
		16		16	14 (87.5)	NA	2 (12.5)
M3	2012	0		16	16 (100)	NA	0
		8		16	16 (100)	NA	0
		32		16	16 (100)	NA	0
M4	2014	0		17	17 (100)	NA	0
		16^*b*^ (arm 1)		17	17 (100)	NA	0
		16^*b*^ (arm 2)		16	15 (93.8)	NA	1 (6.3)

a Total number of animals randomized to treatment.

b Obiltoxaximab products manufactured at two different facilities were tested in arm 1 and arm 2.

c NA, not applicable.

### Treatment administration.

Obiltoxaximab (Elusys Therapeutics) is a chimeric affinity-enhanced monoclonal antibody of the IgG1k isotype purified from cultures of stably transfected nonsecreting NS0 myeloma cells. Obiltoxaximab drug product is formulated in 40 mM histidine, 200 mM sorbitol, and 0.01% polysorbate 80 (Tween 80), pH 5.5, and is provided as a sterile 100 mg/ml solution stored at 2 to 8°C. A single intravenous dose of obiltoxaximab or placebo (sterile 0.9% sodium chloride injection, USP; obtained from B. Braun Medical, Inc., Bethlehem, PA [R1, M1, and M2], Sigma-Aldrich, Irvine, United Kingdom [M3], and Hospira, Inc., Rocky Mount, NC [R2 and M4]) was administered upon detection of either a significant increase in body temperature (SIBT; rabbits only) or serum PA (rabbits and macaques), both of which have been demonstrated to correspond to bacteremia onset (22, 23, 26, 27).

Body temperature was assessed using a subcutaneously implanted programmable temperature transponder injected subcutaneously in each rabbit (IPTT-300; BMDS, Seaford, DE) as described previously (22). A baseline average temperature was calculated for each transponder location from the prechallenge measurements for each rabbit. Following challenge, body temperatures were measured once every hour between 18 and 54 h postchallenge (treatment window), and SIBT was defined as a temperature reading of greater than or equal to a two-standard-deviation increase from baseline temperature either for three consecutive times or for two consecutive times twice. PA was assessed using electrochemiluminescence (termed PA-ECL) to determine the presence or absence of PA in serum above the trigger cut point as previously described (23). This method was used to rapidly assess toxemia as a trigger for treatment. Briefly, serum samples were tested for the presence of PA by a qualitative electrochemiluminescence method every 6 h starting 18 to 24 h after spore exposure. Both a negative control (NC; matched serum sample) and positive control (PC; 1 ng/ml PA for rabbits and 2 ng/ml PA for macaques) were used to monitor the acceptability of the assay. Acceptance criteria were based on <35% coefficient of variation (CV) and the signal-to-noise ratio (S/N ratio; determined as the mean ECL value of the PC divided by the mean ECL value of the NC). For the trigger threshold, the S/N ratio of PC had to be greater than or equal to 1.54 for rabbits and 1.31 for primates.

Levofloxacin (Levaquin oral solution; Ortho-McNeil, Raritan, NJ) or water was administered by gastric intubation once the treatment trigger criteria were met, and 24 h (±1 h) and 48 h (±3 h) later obiltoxaximab or saline was administered within 10 min after administration of the first levofloxacin dose.

### Study conduct.

All studies were conducted at the biosafety level 3 facilities at Battelle Biomedical Research Center, Columbus, Ohio, with the approval of Battelle's Institutional Animal Care and Use Committee and in compliance with FDA Good Laboratory Practice regulations (21 CFR Part 58).

In all treatment studies, animals were observed every 6 h beginning 18 h (rabbits) or 24 h (macaques) following challenge and ending at either 7 days (rabbits) or 8 days (macaques) after the challenge for mortality and outward clinical signs of anthrax disease. After study day 7 in rabbits and study day 8 in macaques, animals were observed twice daily.

### Pharmacodynamic measurements.

Blood samples for pharmacodynamic measurements were taken from a femoral artery or appropriate vein in macaques and from the VAP or medial artery in rabbits. All postchallenge, pretreatment blood samples were collected relative to the median challenge time at the time points stated (±1 h).

During infection, PA83 is cleaved into C-terminal PA63 and the free N-terminal PA20 (28). A sandwich enzyme-linked immunosorbent assay (ELISA) method was validated (29, 30) to quantify PA63 and/or PA83 in rabbit or macaque serum. Obiltoxaximab (Elusys Therapeutics) was utilized as a capture reagent following immobilization on high-binding, 96-well, half-area plates (Corning, Corning, NY). The primary detection reagent was goat anti-PA antisera with horseradish peroxidase-conjugated anti-gamma chain purified secondary antibody (IgG; Invitrogen; Carlsbad, CA) as the reporter and tetramethyl benzidine (TMB; Thermo Fisher; Waltham, MA) as the substrate. The assay detected neither PA20 nor PA bound to serum obiltoxaximab. The assay lower limit of quantitation (LLOQ) was 9.68 ng/ml, and the upper limit of quantitation (ULOQ) was 40,000 ng/ml.

Bacteremia was quantitated by plate culture of blood samples for 16 to 24 h. Fresh blood samples were diluted 1:10 serially, and each dilution was plated in triplicate with an acceptable range between 25 and 250 colony counts per plate. The concentration of samples (CFU/ml) was determined as [(mean number of colonies on three plates) × (total dilution factor)]/(inoculation volume in milliliters).

### Statistical considerations.

The primary endpoint was the survival rate, defined as the percentage of animals alive at the time of scheduled study termination. Study termination occurred on day 28 postchallenge in all studies except for study M4, which terminated on day 28 (50% of animals) or day 56 postchallenge. The survival rate in each of the obiltoxaximab groups was compared to the placebo in the modified intent-to-treat population (all randomized animals bacteremic prior to treatment) using one-sided Boschloo's exact test with a Berger-Boos gamma correction of 0.001 (31). Analyses were conducted with R using the package “exact.” Exact 95% confidence intervals (CI) for differences in survival rates are based on the score statistic (Proc Freq of S SAS, version 9).

The effects of body weight, age, administered dose, and pretreatment bacteremia on survival in studies M1 to M4 were assessed using analysis of variance models fitted to the data with an effect for study. Pairwise comparisons of the studies were made using Tukey's multiple-comparison adjustment procedure. Logistic regression analyses were performed to determine the relationship between survival and pretreatment bacterial and PA levels. Analyses were conducted using SAS, version 9.

### Survival modeling.

The obiltoxaximab dose-response relationship was characterized using a Weibull cure rate model for survival. The effect of obiltoxaximab was entered into the model using an *E*_max_ (maximal effect) model with an exponential effect of log_10_(PTT bacteremia) on logit(*p*_surv_), where PTT indicates prior to treatment and *p*_surv_ is the probability that an animal survives to the end of the study. Survival modeling was performed using a parametric cure rate model and covariates of dose, bacterium levels prior to treatment, and species. The survivor function for the Weibull cure rate model was described by the equation *P*(*T* > *t*) = *p*_surv_ + (1 − *p*_surv_) exp[−(λ*t*)^*a*^], where parameter *T* is the time to death and λ is the rate at which animals die. The shape of the survival curve is determined by the parameter *a*. The survival model also included an *E*_max_ dose response and an exponential effect of log_10_(PTT bacteremia) on logit(*p*_surv_). The parameters *p*_surv_ and λ were modeled as functions of dose and PTT quantitative bacteremia in the following manner: logit(*p*_surv_) = ϴ_0_ − exp[(ϴ_1_ × log_10_(PTT))^ϴ2^] + *E*_max_ × dose/ED_50_ + dose and log(λ) = λ_0_ + λ_1_ × log_10_(PTT), where parameter *E*_max_ is the maximal effect of obiltoxaximab on *p*_surv_ on the logit scale, ϴ_0_ is the baseline logit for *p*_surv_, ϴ_1_ is the rate for log_10_(PTT bacteremia) *p*_surv_, and ϴ_2_ is the exponent for quantitative bacteremia effect. The parameter ED_50_ is the dose (milligrams per kilogram) that is needed to achieve half of the maximal effect of drug on *p*_surv_. The shape parameter (*a*) was not found to depend on any of the covariates that were evaluated. The effect of obiltoxaximab enters into the model only in regard to the probability of survival. Preliminary models demonstrated that obiltoxaximab dose amount did not affect rate of death. The estimated ED_50_ is 1.64 mg/kg (95% confidence interval, 0.515 to 5.22).

## RESULTS

### Therapeutic efficacy of obiltoxaximab.

**FIG 1:**
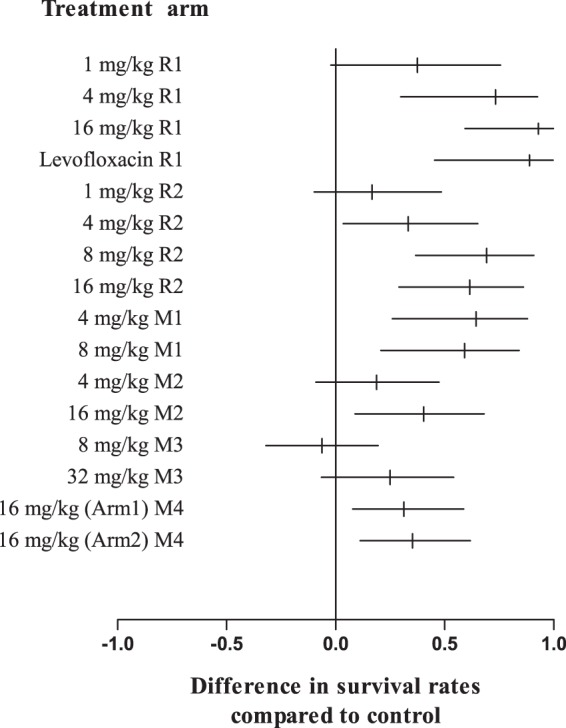
Toxin neutralization with obiltoxaximab confers survival benefit in the treatment of inhalational anthrax. NZW rabbits (studies R1 and R2) and cynomolgus macaques (studies M1 to M4) were aerosol challenged with targeted 200 LD_50_ of B. anthracis spores. Treatment was administered at the indicated doses following the first detection of circulating PA by PA-ECL (all studies) or significant increase in body temperature (rabbits only). Forest plot represents differences between survival proportions in placebo (control) and obiltoxaximab treatment groups as vertical lines with corresponding confidence intervals (horizontal lines). Only animals bacteremic at the time of treatment were included in the analyses.

Obiltoxaximab administration led to improved survival compared to placebo at doses ranging from 1 to 32 mg/kg in multiple studies in rabbits and cynomolgus macaques (Table 2). Treatment was triggered by PA-ECL in 35.3 to 66.7% and 14.3 to 35.7% of rabbits in studies R1 and R2, respectively, with the rest of the animals triggering by SIBT (Table 1). In contrast, the majority (85.7 to 100%) of macaques across all studies were triggered by PA-ECL (Table 1). Significantly improved survival was consistently observed with a single 16 mg/kg obiltoxaximab dose compared to placebo. Rabbits treated with 16 mg/kg obiltoxaximab had survival rates of 93% and 62% compared with a survival rate of 0% in placebo-treated animals (*P* = 0.0010 and *P* = 0.0013, respectively), and the survival rate in study R1 (93%) was comparable to the survival rate for rabbits treated with levofloxacin (88.9%). Macaques treated with 16 mg/kg obiltoxaximab had survival rates of 31%, 35%, and 47% compared with survival rates of 0%, 0%, and 6.3% in placebo-treated animals (*P* = 0.0085, *P* = 0.0053, and *P* = 0.0068, respectively), and a dose increase to 32 mg/kg did not provide additional benefit (Fig. 1). Significant increases in survival were also seen with 8 mg/kg obiltoxaximab compared to placebo in study R2 in rabbits (69% versus 0%, respectively; *P* = 0.0011) and study M1 in macaques (73% versus 14%, respectively; *P* = 0.0017), but not in study M3 (6.3% versus 12.5%, respectively).

**TABLE 2:** Survival outcomes in obiltoxaximab treatment studies

Treatment dose (mg/kg)	% Survival^*b*^ (no. surviving/total no.), *P* value, for study:						
	R1	R2	M1	M2	M3	M4^*a*^	
						Arm 1	Arm 2
Placebo	0 (0/9)^*d*^, NA	0 (0/13), NA	14.3 (2/14)	6.3 (1/16)	12.5 (2/16)	0 (0/17)	
1	37.5 (3/8)^*d*^, 0.0327	16.7 (2/12), 0.1187	—	—	—	—	—
4	73.3 (11/15), 0.0012^*c*^	33.3 (4/12), 0.0232^*c*^	78.6 (11/14), 0.0015^*c*^	25.0 (4/16), 0.1074	—	—	—
8	—	69.2 (9/13), 0.0011^*c*^	73.3 (11/15), 0.0017^*c*^	—	6.3 (1/16), 0.8038	—	—
16	92.9 (13/14), 0.0010^*c*^	61.5 (8/13), 0.0013^*c*^	—	46.7 (7/15), 0.0068^*c*^	—	31.3 (5/16), 0.0085^*c*^	35.3 (6/17), 0.0055^*c*^
32	—	—	—	—	37.5 (6/16), 0.0599	—	—
Levofloxacin	88.9 (8/9), 0.0011^*c*^	—	—	—	—	—	—

a Obiltoxaximab products manufactured at two different facilities were tested in arm 1 and arm 2.

b Only animals that were bacteremic prior to treatment were included in the analyses. NA, not applicable; —, dose not tested.

c Statistical significance at the 0.025 level.

d One animal was inadvertently dosed with levofloxacin and excluded from analyses.

### Pharmacologic activity of obiltoxaximab.

**FIG 2:**
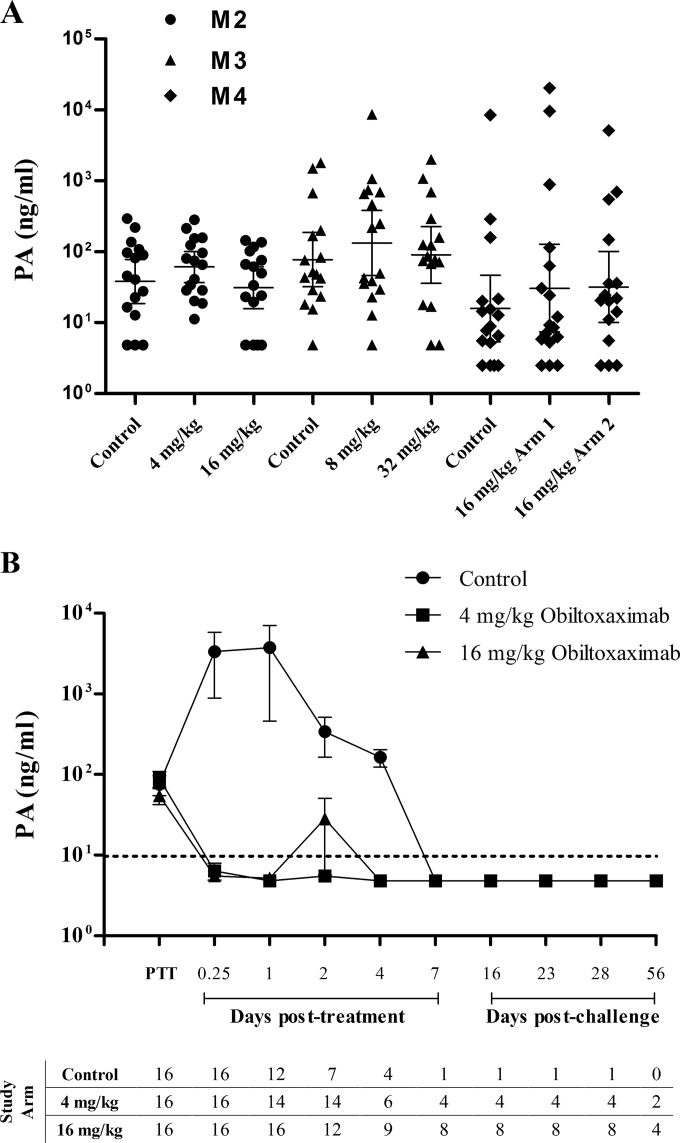
Neutralization of circulating PA following obiltoxaximab administration. Cynomolgus macaques in studies M2 to M4 were aerosol challenged with targeted 200 LD_50_ of B. anthracis spores, and placebo or obiltoxaximab was administered following the first detection of circulating PA by PA-ECL. Peripheral blood samples were collected immediately prior to treatment (PTT) or at the indicated times posttreatment or postchallenge for the assessment of circulating free PA. (A) Shown are individual animal values for free PA values obtained immediately prior to treatment in each dose group. Horizontal lines indicate geometric means and 95% CI. Free PA was not measured in study M1. (B) Shown are means and 95% CI for free PA levels in samples collected at each indicated time point in study M2. Numbers of animals surviving to each sample collection are indicated on the bottom. The single control survivor and 50% of obiltoxaximab-treated survivors were sacrificed on study day 28. Terminal samples are not included on the graph. Upper limit of quantitation (ULOQ) and lower limit of quantitation (LLOQ) for free PA assay were 40,0000 and 9.68 ng/ml, respectively; LLOQ is indicated by the dotted line. For statistical computations, PA levels below the limit of detection were replaced with 4.84 ng/ml (1/2 LLOQ).

Pharmacologic activity of obiltoxaximab was measured by its ability to reduce circulating PA levels in studies R2 and M2 to M4. In study R2, treatment was triggered by significant elevation in temperature in 75% of animals, and circulating PA was detected in 8.5% of rabbits. We therefore focused analyses on cynomolgus macaque studies. In macaque studies, a range of pretreatment PA levels was observed among individual animals and across studies (Fig. 2A), and 93% (M1), 96% (M2), 100% (M3), and 98% (M4) of animals received treatment based on PA elevation. Administration of 4 to 32 mg/kg obiltoxaximab reduced serum PA concentrations to below the lower limit of quantitation at the earliest posttreatment time point examined (15 min to 24 h) (Fig. 2B and data not shown). PA was not measured in study M1. All animals treated with obiltoxaximab showed a similar decrease in PA (Fig. 2B and data not shown), demonstrating that obiltoxaximab exerted the expected pharmacological effect. In contrast, PA concentrations and the number of animals with a PA concentration above the limit of quantitation in the placebo group increased until the animals succumbed in all macaque studies (Fig. 2B and data not shown). Comparable results were obtained in study R2 (data not shown).

### Disease spectrum in cynomolgus macaque studies.

**FIG 3:**
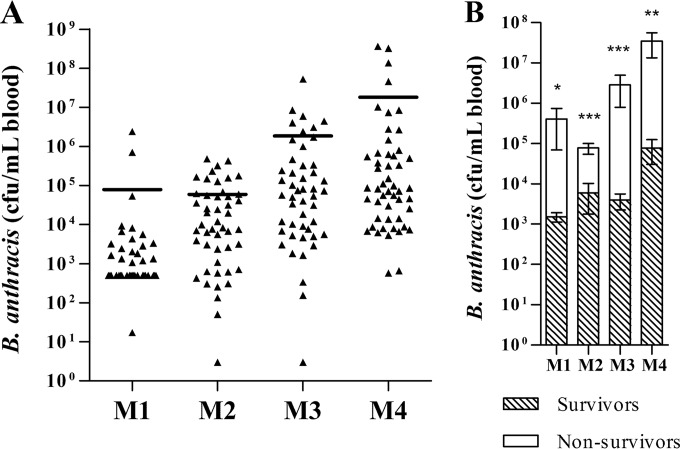
PTT bacteremia in survivors and nonsurvivors of cynomolgus macaque treatment studies. Cynomolgus macaques in studies M1 to M4 were aerosol challenged with targeted 200 LD_50_ of B. anthracis spores, and placebo or obiltoxaximab was administered following the first detection of circulating PA by PA-ECL. Peripheral blood samples were collected immediately PTT for quantitative assessment of bacteremia. (A) Shown are geometric means and individual animal values in each study. Both placebo- and obiltoxaximab-treated animals were included in the analyses. (B) Shown are means and SEM for bacteremia levels in survivors (shaded bars) and nonsurvivors (white bars) obtained prior to treatment for each group that received obiltoxaximab. The limit of detection (LOD) was 33 CFU/ml in M1 and 3 CFU/ml in M2 to M4; the limit of quantification (LOQ) was 1,000 CFU/ml in M1 and 100 CFU/ml in M2 to M4. *, *P* < 0.05; **, *P* < 0.01; ***, *P* < 0.001 compared to the control (Mann-Whitney test). Bacteremia levels below the limit of detection were replaced with 1/2 LOD, and levels above limits of detection but below limits of quantitation were replaced with 1/2 LOQ.

We examined bacteremia levels immediately before treatment to determine if this predicted the treatment response. In cynomolgus macaques, the highest levels of pretreatment bacteremia were observed in studies M3 and M4, with individual animal counts reaching 10^7^ to 10^8^ CFU/ml (Fig. 3A). Bacteremia levels at the time of treatment increased exponentially across the studies in a manner that correlated inversely with survival (Tables 2 and 3). In macaque studies, mean pretreatment bacteremia was significantly higher in animals that ultimately succumbed to infection, and only one survivor was documented with bacteremia levels exceeding 10^5^ CFU/ml (Fig. 3B).

**TABLE 3:** PTT bacteremia levels in cynomolgus macaque monotherapy studies

Study	No. of animals			Quantitative levels of blood bacteremia (CFU/ml)	
	Total	<LLOQ	<LOD	Geometric mean (% CV)	95% CI
M1	43	20	2	1,670 (1,395)	822, 3,380
M2^*a*^	48	1	1	8,270 (2,923)	3,890, 17,600
M3^*b*^	48	0	1	55,700 (13,881)	22,400, 139,000
M4^*c*^	50	0	0	150,000 (11,051)	62,700, 358,000

a Tukey's pairwise comparisons with the following studies were significant at the 0.05 level: M1 (ratio of geometric study means, 4.96).

b Tukey's pairwise comparisons with the following studies were significant at the 0.05 level: M1 (ratio of geometric study means, 33.43) and M2 (ratio of geometric study means, 6.74).

c Tukey's pairwise comparisons with the following studies were significant at the 0.05 level: M1 (ratio of geometric study means, 89.87) and M2 (ratio of geometric study means, 18.13).

### Survival covariates.

To understand variability in survival results across the four primate studies, effects of pretreatment bacteremia, weight, age, and challenge dose were analyzed. Although there were significant differences between age, weight, and challenge dose variables of animals across studies (Tables 4 to 6), the differences did not correlate with survival outcomes.

**TABLE 4:** Prior-to-challenge body weight in cynomolgus macaque monotherapy studies

Study	*n*	Weight at challenge (kg)	
		Mean (SD)	95% CI
M1^*a*^	43	3.30 (0.61)	3.11, 3.49
M2	48	2.82 (0.24)	2.75, 2.89
M3^*b*^	48	3.90 (0.60)	3.72, 4.07
M4	50	2.88 (0.43)	2.76, 3.00

a Tukey's pairwise comparisons with the following studies were significant at the 0.05 level: M2 (the difference of study means, 0.48) and M4 (the difference of study means, 0.42).

b Tukey's pairwise comparisons with the following studies were significant at the 0.05 level: M1 (the difference of study means, 0.6), M2 (the difference of study means, 1.08), and M4 (the difference of study means, 1.02).

**TABLE 5:** Prior-to-challenge age in cynomolgus macaque monotherapy studies

Study	*n*	Age at challenge (yr)	
		Mean (SD)	95% CI
M1^*a*^	43	3.74 (0.57)	3.56, 3.92
M2	48	3.05 (0.19)	2.99, 3.10
M3^*b*^	48	4.98 (0.37)	4.87, 5.08
M4^*c*^	50	3.38 (0.44)	3.25, 3.50

a Tukey's pairwise comparisons with the following studies were significant at the 0.05 level: M2 (the difference of study means, 0.7) and M4 (the difference of study means, 0.36).

b Tukey's pairwise comparisons with the following studies were significant at the 0.05 level: M1 (the difference of study means, 1.23), M2 (the difference of study means, 1.93), and M4 (the difference of study means, 1.6).

c Tukey's pairwise comparisons with the following studies were significant at the 0.05 level: M2 (the difference of study means, 0.33).

**TABLE 6:** Challenge dose in cynomolgus macaque monotherapy studies

Study	*n*	Challenge dose (LD_50_ CFU/animal)	
		Geometric mean (%CV)	95% CI
M1	43	191 (30.06)	175, 209
M2	48	208 (20.59)	196, 221
M3^*a*^	48	280 (25.04)	261, 301
M4^*b*^	50	252 (20.10)	238, 267

a Tukey's pairwise comparisons with the following studies were significant at the 0.05 level: M1 (ratio of geometric study means, 1.47) and M2 (ratio of geometric study means, 1.35).

b Tukey's pairwise comparisons with the following studies were significant at the 0.05 level: M1 (ratio of geometric study means, 1.32) and M2 (ratio of geometric study means, 1.21).

**FIG 4:**
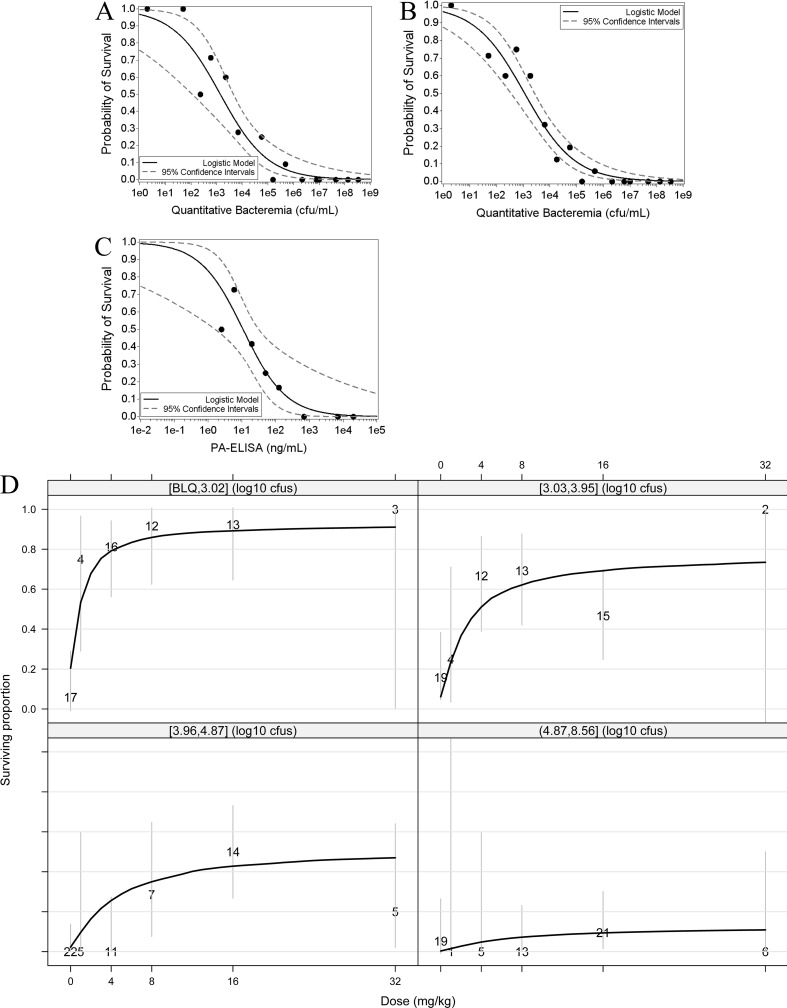
Levels of bacteremia and toxemia at the time of toxin neutralization are prognostic of survival following antitoxin monotherapy. Shown are logistic regression analyses describing the relationship between survival and bacteremia (A and B) or survival and toxemia (C) for animals in studies M1 to M4 treated with obiltoxaximab at 16 mg/kg (A and C) or all doses (4 to 32 mg/kg) (B). Filled circles indicate the proportion of animals that survived within each half-log bin. Control animals were not included in the analyses shown in panels A to C. Panels A and B include data from studies M1 to M4, and panel C includes data from studies M2 to M4. (D) Shown are dose-response relationships after stratifying by level of pretreatment bacteremia. The four quadrants represent quartiles of the PTT bacteremia distribution across 5 studies (rabbit study R1 and cynomolgus macaque studies M1 to M4). Bacteremia levels in each quartile are shown on top of each quadrant. Numbers on the plot indicate the total number of animals comprising each observed mean data point, and the solid black line represents the prediction based on modeling of survival data. Gray vertical lines represent 95% confidence intervals. CFU, CFU/ml; BLQ, below the limit of quantitation.

The impact of pretreatment bacteremia on probability of survival following treatment was explored through logistic regression analyses. An inverse relationship between bacteremia levels prior to treatment and survival probabilities was observed (Fig. 4A and B), with a 50% survival probability projected at 10^3^ CFU/ml irrespective of whether analyses included all treatment doses (Fig. 4B) or only the 16 mg/kg dose (Fig. 4A). The relationship between survival and pretreatment toxemia levels was also explored (Fig. 4C). Regression analyses predict that administration of obiltoxaximab as a monotherapy confers a survival benefit up to a bacterial level of 10^5^ CFU/ml and a PA level of 100 ng/ml (10% and 20% survival, respectively, at the indicated threshold levels).

Levels of pretreatment bacteremia associated with different survival probabilities were further characterized with combined rabbit and macaque data. Bacteremia data were collected for rabbit study R2 only with geometric mean bacteremia across treatment arms of 1,318 CFU/ml (CI, 557, 3,116). Model predictions for the relationship between survival outcomes, dose, and pretreatment bacteremia distribution were determined separately for bacteremia quartiles (Fig. 4D). Maximum expected survival was approximately 30%, with bacterial levels in the range of 10^4^ to 10^5^ CFU/ml (comparable to those observed in studies M3 and M4 in macaques) and approximately 80% with bacterial levels up to 10^3^ CFU/ml (comparable to those observed in study M1 in macaques) (Table 2). There was a negligible probability of survival at bacteria levels of ≥10^5^ CFU/ml under conditions of obiltoxaximab monotherapy.

## DISCUSSION

Inhalational anthrax due to intentional or accidental exposure to spores remains a significant concern, and there is a recognized need for adjunctive antitoxin therapies (14, 15). Because human anthrax clinical trials are not feasible, antitoxin efficacy in humans must be extrapolated from animal studies. Here, efficacy of a toxin-neutralizing monoclonal antibody, obiltoxaximab, was investigated in a series of studies conducted in two well-characterized animal models of inhalational anthrax, NZW rabbit and cynomolgus macaque models (22, 23), which have been accepted as a basis of approval under the FDA's Animal Rule (20). Our studies show that a single 16 mg/kg obiltoxaximab dose effectively neutralized PA and improved survival in multiple studies in rabbits and macaques with systemic inhalational anthrax. Obiltoxaximab administration was highly protective when levels of bacteremia were low (macaque study M1). Statistically significant and clinically meaningful survival was also achieved with obiltoxaximab given as monotherapy to animals with pretreatment bacteremia indicative of advanced systemic disease (such as that observed in macaque study M4) and, as may be expected, in inhalational anthrax patients coming to the hospital after protracted prodromal illness. The high efficacy seen with obiltoxaximab monotherapy in study M1 underscores that improved survival outcomes could be achieved with prompt identification and treatment of infected patients to mitigate deleterious effects of toxin at disease onset.

In all studies, obiltoxaximab rapidly neutralized serum PA immediately following administration to below the limit of quantification with comparable reduction in survivors and nonsurvivors. These results show that obiltoxaximab had the predicted pharmacologic effect in all animals and suggest that death in animals with more advanced systemic disease was likely because of direct and irreversible toxin-mediated damage, such as organ and hemodynamic impairment, as well as systemic bacterial dissemination and infection of peripheral tissues prior to treatment. This is supported by the observation that survival was inversely correlated with disease severity (pretreatment bacteria and toxemia levels). Our results also highlight that efficacy analyses based on multiple studies with a spectrum of disease heterogeneity reflective of the likely clinical scenario provide a more complete understanding of antitoxin effectiveness.

Model predictions for the relationship between survival outcomes and pretreatment bacteremia defined the window of effectiveness of obiltoxaximab monotherapy and suggested that when bacteremia levels exceed 10^5^ CFU/ml, treatment prognosis for monotherapy is poor. Experimental data in animal models suggest that a similar efficacy threshold exists for antibiotics such as doxycycline and ciprofloxacin administered as monotherapy, with efficacy significantly reduced when levels of bacteria in the blood exceed levels of 10^5^ CFU/ml (32). In contrast, coadministration of antitoxins with antibiotics under conditions when antibiotics alone were partially effective improved survival rates, as demonstrated for obiltoxaximab (33) and other antitoxins (34, 35).

Survival outcomes in individual animals potentially can be impacted by preexisting antibodies to PA, a concern particularly relevant for cynomolgus macaque studies where a pathogen-free environment prior to placement in the study could not be definitively ascertained. Lack of preexisting antibodies to PA was confirmed in one study, M4, but not in studies M1, M2, or M3. However, we believe that application of strict randomization procedures allowed us to generate impartial and valid survival analyses across all studies.

Another monoclonal antibody, raxibacumab, was approved by the FDA in 2012 for the treatment of inhalational anthrax (20). Both obiltoxaximab and raxibacumab are indicated for the treatment of inhalational anthrax in conjunction with antibiotics and for prophylaxis of anthrax when alternative therapies are not available or appropriate and are being procured for the Strategic National Stockpile. Obiltoxaximab, an IgG1 antibody, was derived from murine monoclonal antibody 14B7 (36) through modifications that included affinity enhancement, humanization, and deimmunization, and it has a *K_D_* (equilibrium dissociation constant) of 0.33 nM (18). Raxibacumab, a human IgG1 antibody isolated through phage display (14), binds PA with an affinity of 2.78 nM (19), approximately one log lower than that of obiltoxaximab. Both antibodies bind domain 4 of PA (14); however, epitope specificities of these antibodies cannot be compared, because no information is available regarding the raxibacumab binding site. Based on available information, raxibacumab efficacy had been tested in one treatment study in cynomolgus macaques (19 and http://www.accessdata.fda.gov/drugsatfda_docs/nda/2012/125349Orig1s000MedR.pdf), conducted in a time frame similar to that of obiltoxaximab study M1 (2007 to 2008 for raxibacumab [http://www.accessdata.fda.gov/drugsatfda_docs/nda/2012/125349Orig1s000MedR.pdf] and 2009 for obiltoxaximab) and at the same testing site. Results of the raxibacumab study (50% and 64% for 20 mg/kg and 40 mg/kg doses, respectively) were comparable to the survival outcomes observed in obiltoxaximab study M1 (78.6% and 73.3% for 4 mg/kg and 8 mg/kg doses, respectively). In two raxibacumab treatment studies conducted in NZW rabbits, efficacy at the highest tested dose was consistently higher for obiltoxaximab than raxibacumab (92.9% and 61.5% versus 44.4% and 45.8%, respectively). It should be noted that bacteremia was not measured quantitatively in the raxibacumab studies, and disease severity at the time of treatment cannot be assessed. Because pretreatment bacteremia is a significant covariate of survival, efficacies of obiltoxaximab and raxibacumab cannot be directly compared in the absence of a head-to-head study. A head-to-head study to investigate comparative efficacies in primates may not, however, be feasible considering the large numbers of animals required.

Overall, data presented here show that obiltoxaximab provides a significant survival benefit in the treatment of inhalational anthrax, with consistent and robust efficacy observed with a single 16 mg/kg dose administered to symptomatic animals and proven efficacy under conditions of high disease severity in the cynomolgus macaque model. Obiltoxaximab effectiveness was established in multiple studies in two animal models that meet the requirements of the Animal Rule and in the absence of concomitant medications (e.g., antibiotics) and supportive care interventions that would typically be administered in a clinical setting. A range of survival benefits were observed across cynomolgus macaque studies, and differences in survival rates were attributed to differences in the severity of anthrax infection immediately prior to treatment as measured by levels of toxemia and bacteremia. We propose that concomitant administration of obiltoxaximab with antibiotics to patients with inhalational anthrax will mitigate toxemia and extend the window of treatment effectiveness, providing significant improvement in survival at both early and advanced stages of disease. Obiltoxaximab approval addresses public health needs by providing the U.S. government with a second supplier of a monoclonal antibody anthrax antitoxin, ensuring redundancy in supply and decreasing risks associated with relying on a sole source of monoclonal antitoxin.
